# Antigonococcal Activity and Cytotoxicity Screening of Three *Ziziphus* Species Indigenous to South Africa

**DOI:** 10.1002/cbdv.71477

**Published:** 2026-07-06

**Authors:** Mcebisi J. Mabuza, Marcel Kaiser, Thilivhali E. Tshikalange, Abdullahi A. Yusuf, Mahwahwatse J. Bapela

**Affiliations:** ^1^ Department of Plant and Soil Sciences University of Pretoria Pretoria South Africa; ^2^ Department of Life and Consumer Sciences University of South Africa Florida South Africa; ^3^ Swiss Tropical and Public Health Institute Allschwil Switzerland; ^4^ Department of Zoology and Entomology University of Pretoria Hatfield South Africa

**Keywords:** antigonococcal activity, cytotoxicity, *Neisseria gonorrhoeae*, South Africa, *Ziziphus*

## Abstract

Despite the extensive use of *Ziziphus* species in South African traditional medicine to treat various ailments, they remain poorly studied. Therefore, this study aimed to scientifically validate the ethnomedicinal use of *Z. mucronata* Willd., *Z. rivularis* Codd, and *Z. zeyheriana* Sond. in the treatment of gonorrhea. Their roots, stem bark, and leaves were sequentially extracted using five different solvents. The extracts were evaluated against *Neisseria gonorrhoeae* using the broth microdilution assay and cytotoxicity in rat skeletal (L6) myoblast cell lines. Of the 48 successive extracts evaluated, 12.5% exhibited high antigonococcal activity (MIC < 1 mg/mL), 33.3% showed moderate activity (MIC 1–6.25 mg/mL), and 54.1% were inactive (MIC > 6.25 mg/mL). Furthermore, the extracts displayed low cytotoxicity profiles, with 81% exhibiting no cytotoxicity (CC_50_ > 0.05 mg/mL) and the remaining 19% cytotoxic (CC_50_ < 0.05 mg/mL). Phytoconstituents tentatively identified and putatively attributed to the observed antigonococcal activity were *n*‐hexadecanoic acid, phytol, and betulin. These have not been previously studied against *N. gonorrhoeae*; further bioactivity screening in both in vivo and in vitro models is still needed to confirm their antigonococcal activity. Future research should focus on isolating phytoconstituents and testing the antigonococcal activity in vitro and in vivo.

## Graphical abstract

*Ziziphus* species in South Africa are widely used to treat gonorrhea; however, to this day, there has been no scientific validation of their ethnomedicinal use. This study tentatively identified phytol, betulin, and hexadecanoic acid to be linked to the observed antigonococcal activity. Further isolation and bioactivity testing in vivo are needed to establish their therapeutic potential.

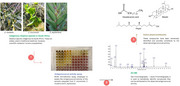

## 1 Introduction

*Neisseria gonorrhoeae* remains a significant and evolving bacterial threat, disproportionately affecting the African continent. A high number of cases have been reported among sexually active populations between the ages of 15 and 49 years [1]. Globally, approximately 82 million new gonorrhea infections are reported annually, with a significant increase in infections among men who have sex with men [2]. This trend is particularly pronounced in sub‐Saharan Africa, where South Africa bears the largest share of the burden [3, 4]. Consequently, the gonorrhea burden is further exacerbated by the syndemic relationship between gonorrhea and human immunodeficiency virus (HIV), which complicates the clinical management of gonorrhea [5]. This places additional strain on already constrained healthcare systems and treatment strategies for better managing these diseases. Although effective gonorrhea chemotherapeutic regimens are available, access, especially in low– and middle– income regions in Africa, is uneven [6, 7]. This unequal access is compounded by reliance on a limited number of antimicrobial drugs, which exerts selective pressure on *N. gonorrhoeae* populations. There is currently no approved vaccine to treat gonorrhea, making antibiotics the primary treatment option. This highlights the necessity of discovering novel drug leads with different chemical structures and varying modes of action [8, 9, 10, 11].

The emergence of widespread antimicrobial resistance (AMR) to prescribed antibiotic regimens threatens the efficacy of currently available drugs, leaving a few treatment alternatives in the antigonococcal therapeutic pool [12]. While AMR was initially associated with mono‐chemotherapeutics, recent findings suggest that *N. gonorrhoeae* has also developed resistance to dual‐combination treatments, thereby worsening the gonorrhea burden [13, 14, 15]. In response to this, new effective antibiotics with different modes of action and chemical scaffolds need to be developed [16, 17]. It is against this backdrop that medicinal plants are resorted to as potential sources of new chemotherapeutic agents. These have the potential to provide chemical compounds for the development of novel antibiotics [18]. Although no commercial antibiotics have been directly derived from plants, the extensive chemical constituents found in them are promising candidates for antibiotic discovery and further development [19]. Examining medicinal plants may lead to the development of next‐generation antibiotics with diverse chemical scaffolds that effectively target *N. gonorrhoeae*.

Examples of plants with known antimicrobial properties include species of the cosmopolitan genus *Ziziphus*. These hold great significance in South African traditional medicine, with *Z. mucronata* Willd., *Z. rivularis* Codd, and *Z. zeyheriana* Sond. having been used for generations. The ethnomedicinal significance of these species has been observed in their ability to treat fevers, diabetes, malaria, swollen glands, respiratory ailments, dysentery, septic wounds, and STIs, including gonorrhea [20, 21, 22, 23, 24, 25, 26]. Despite their traditional use to treat various STIs, they have not been extensively evaluated for their antigonococcal activity or cytotoxicity. Furthermore, information on their chemical profiling and isolated phytoconstituents has not been investigated in depth. The aim of this study was therefore to investigate the in vitro antigonococcal activity and cytotoxicity of *Z. mucronata*, *Z. rivularis*, and *Z. zeyheriana*. In addition, it sought to tentatively identify antigonococcal chemical classes and phytoconstituents using ^1^H NMR‐based metabolomics and GC–MS, respectively.

## 2 Results and Discussion

The cytotoxicity screening of all 48 successive extracts revealed that 27% were non‐cytotoxic, while 54% exhibited moderate cytotoxicity, and 19% were highly cytotoxic to L6 rat skeletal cells (Table 1). A comparative analysis of the different plant parts highlighted that the cytotoxicity observed in the leaf extracts was very low, while the roots were moderately cytotoxic, and the stem bark extracts were highly cytotoxic. Notably, as previously reported, the leaf extracts of *Z. rivularis* demonstrated the most favorable cytotoxicity profile, achieving CC_50_> 0.05 mg/mL [27]. Hence, *Ziziphus* species are generally regarded as non‐cytotoxic to various human cell lines, and the findings of this study support this observation [28]. Although there was no established correlation between solvent polarity and cytotoxicity, it was noted that cytotoxicity was limited to extracts that were associated with the use of dichloromethane, ethyl acetate, or a combination of ethyl acetate and methanol (1:1; *v/v*) as extraction solvents. The decoctions showed limited cytotoxicity on rat myoblast cell lines, except *Z. rivularis* leaf and *Z. zeyheriana* root extracts, which reported CC_50_ values below 0.05 mg/mL. Typically, samples with low cytotoxicity are favored for further exploration in drug discovery and development. However, it's crucial to recognize that in vitro tests alone cannot predict the clinical safety of any crude extract, and thus in vivo evaluations are necessary to determine the clinical safety of these plant extracts [29, 30].

**TABLE 1:** The minimum inhibitory concentration (MIC) values of South African *Ziziphus* species against *N. gonorrhoeae* (ATCC 49226) and mammalian L‐6 cells (CC_50_). The indicated MIC and CC_50_ values represent the means of three independent assays conducted in triplicate (*n* = 3).

Plant (parts)	Sample ID (solvents)	Yield (%)	MIC (mg/mL)^a^	CC_50_ (mg/mL)^b^	Selectivity index (SI)
*Z. mucronata* (leaf)	ZML1 (*n*‐hexane)	1.22	12.5 ± 2.1	>0.1	ND
	ZML2 (DCM)	3.38	3.13 ± 1.1	0.059 ± 2.4	0.02
	ZML3 (EtOAc)	2.49	3.13 ± 0.2	0.069 ± 0.7	0.02
	ZML4 (EtOAc:MeOH (1:1))	11.97	1.56 ± 3.2	>100	ND
	ZML5 (MeOH)	22.10	12.5 ± 0.7	0.064 ± 4.1	0.01
	ZML6 (H_2_O)^c^	3.40	>12.5	0.080 ± 1.1	ND
*Z. mucronata* (stem bark)	ZMS1 (*n*‐hexane)	0.16	1.56 ± 0.6	0.022 ± 0.9	0.01
	ZMS2 (DCM)	0.54	>12.5	0.016 ± 0.1	ND
	ZMS3 (EtOAc)	0.13	12.5 ± 0.4	0.020 ± 2.5	0.001
	ZMS4 (EtOAc:MeOH (1:1))	7.33	3.13 ± 2.2	0.054 ± 3.7	0.02
	ZMS5 (MeOH)	6.03	6.25 ± 0.7	0.051 ± 2.1	0.01
	ZMS6 (H_2_O)^c^	7.17	12.5 ± 0.9	0.066 ± 17.9	0.01
*Z. mucronata* (root bark)	ZMR1 (*n*‐hexane)	1.20	1.56 ± 2.6	0.055 ± 6.6	0.04
	ZMR2 (DCM)	0.84	6.25 ± 0.8	0.024 ± 4.1	0.004
	ZMR3 (EtOAc)	0.89	6.25 ± 2.1	0.07 ± 17.6	0.01
	ZMR4 (EtOAc:MeOH (1:1))	4.83	6.25 ± 1.1	0.050 ± 3.5	0.01
	ZMR5 (MeOH)	10.69	>12.5	0.050 ± 5.1	ND
	ZMR6 (H_2_O)^c^	0.98	>12.5	0.053 ± 0.1	ND
*Z. rivularis* (leaf)	**ZRL1 (*n*‐hexane)**	**1.05**	**0.39 ± 0.2**	**>0.1**	**ND**
	**ZRL2 (DCM)**	**5.31**	**0.2 ± 04**	**>0.1**	**ND**
	**ZRL3 (EtOAc)**	**0.79**	**0.2 ± 0.2**	**>0.1**	**ND**
	**ZRL4 (EtOAc:MeOH (1:1))**	**27.94**	**0.39 ± 0.9**	**0.050 ± 1.8**	**0.13**
	**ZRL5 (MeOH)**	**8.88**	**0.78 ± 0.3**	**0.050 ± 2.4**	**0.06**
	ZRL6 (H_2_O)^c^	0.21	>12.5	0.046 ± 3.1	ND
*Z. rivularis* (stem bark)	ZRS1 (*n*‐hexane)	0.19	6.25 ± 1.5	>0.1	ND
	ZRS2 (DCM)	0.40	6.25 ± 2.2	>0.1	ND
	ZRS3 (EtOAc)*	0.17	0.39 ± 0.1	0.062 ± 11.3	0.16
	ZRS4 (EtOAc:MeOH (1:1))	0.37	3.13 ± 1.1	0.073 ± 21.8	0.02
	ZRS5 (MeOH)	0.36	3.13 ± 2.9	0.053 ± 7.1	0.02
	ZRS6 (H_2_O)^c^	2.31	6.25 ± 0.2	>0.1	ND
*Z. rivularis* (root bark)	ZRR1 (*n*‐hexane)	0.05	6.25 ± 0.9	>0.1	ND
	ZRR2 (DCM)	0.06	6.25 ± 2.1	>0.1	ND
	ZRR3 (EtOAc)	0.32	6.25 ± 4.5	>100	ND
	ZRR4 (EtOAc:MeOH (1:1))	6.98	6.25 ± 0.6	0.052 ± 2.5	0.01
	ZRR5 (MeOH)	7.37	3.13 ± 1.8	0.068 ± 26.8	0.02
	ZRR6 (H_2_O)^c^	4.83	6.25 ± 3.8	>0.1	ND
*Z. zeyheriana* (stem)	ZZS1 (*n*‐hexane)	0.16	6.25 ± 2.3	0.057 ± 10.4	0.01
	ZZS2 (DCM)	0.47	6.25 ± 4.3	0.058 ± 1.3	0.01
	ZZS3 (EtOAc)	0.21	3.13 ± 0.2	0.067 ± 8.3	0.02
	ZZS4 (EtOAc:MeOH (1:1))	2.95	1.56 ± 0.7	0.047 ± 0.4	0.03
	ZZS5 (MeOH)	1.80	3.13 ± 0.4	0.062 ± 17.4	0.02
	ZZS6 (H_2_O)^c^	1.04	12.5 ± 1.2	>0.1	ND
*Z. zeyheriana* (root)	ZZR1 (*n*‐hexane)	0.19	6.25 ± 2.9	0.055 ± 6.2	0.01
	ZZR2 (DCM)	0.78	1.56 ± 4.2	0.019 ± 0.1	0.01
	**ZZR3 (EtOAc)**	**0.43**	**0.2 ± 0.3**	**0.016 ± 0.4**	**0.08**
	ZZR4 (EtOAc:MeOH (1:1))	5.97	1.56 ± 0.6	0.048 ± 0.9	0.03
	ZZR5 (methanol)	5.74	1.56 ± 1.6	0.066 ± 0.2	0.04
	ZZR6 (H_2_O)^c^	1.21	3.13 ± 2.6	0.046 ± 0.5	0.01
10% DMSO^d^			12.5		
Ciprofloxacin^e^			0.09		
Podophyllotoxin^f^				0.008	

*Note*: Samples with high antigonococcal activity (bold).

Abbreviations: ND, not determined; DCM, dichloromethane.

^a^ *N. gonorrhoeae*.

^b^ Rat skeletal myoblast human cell lines (L‐6 cell lines).

^c^ Decoction.

^d^ Microdilution assay negative control.

^e^ Microdilution assay positive control.

^f^ Cytotoxicity assay control.

Screening of the extracts against *N. gonorrhoeae* (ATCC 49226) showed a range of antigonococcal activity (Table 1). Of the 48 extracts tested, 12.5% showed high activity (MIC < 1 mg/mL), 33.3% were moderately active (1 mg/mL ≤ MIC ≤ 6.25 mg/mL), and the majority (54.1%) were inactive (MIC > 6.25 mg/mL). Although a smaller proportion of the extracts exhibited limited antigonococcal activity, it is a common occurrence, especially among crude plant extracts. Their complex metabolome can mask or interfere with the activity of bioactive compounds [16, 17]. When comparing the observed antigonococcal activity across the different plant parts, the leaf extracts, particularly those from *Z. rivularis*, exhibited the highest antigonococcal activity. The hexane (ZRL1), dichloromethane (ZRL2), ethyl acetate (ZRL3), ethyl acetate:methanol (1:1; *v/v*) (ZRL4), and methanol (ZRL5) displayed the strongest inhibitory effects, with MIC values of 0.39, 0.2, 0.2, 0.39, and 0.78 mg/mL, respectively. This consistent antigonococcal bioactivity across extracts of different polarities may suggest a broad spectrum of chemical constituents that span a wide polarity range in the leaf extracts. In contrast, the root extracts were relatively poorly active, except for the ethyl acetate root extract of *Z. zeyheriana* (ZZR3), which showed high activity (MIC = 0.2 mg/mL). This observation indicates that although the roots may contain some bioactive constituents, their distribution and relative concentrations differ from those in the leaves. The higher bioactivity observed in the leaves can be attributed to the accumulation of defensive secondary metabolites in the plant's aerial parts, which are mostly exposed to environmental stresses compared to the roots and stem bark.

Furthermore, the current findings revealed that non‐polar and semi‐polar extracts demonstrate enriched antigonococcal bioactivity compared to polar extracts. This observed trend may suggest that the larger proportion of the bioactive constituents is likely lipophilic in nature, potentially including chemical classes like terpenoids and some alkaloids known to elicit antimicrobial effects [31, 32]. The low antigonococcal activity observed in most polar extracts further supports the idea that highly polar extracts do not elicit the desired antigonococal activity [33]. This is corroborated by the majority of the tested decoctions, which were inactive, except for the root extract of *Z. zeyheriana*, which demonstrated activity with an MIC value of 3.13 mg/mL. Consequently, these findings contrast with previous observations by Erasmus et al., where antigonococcal activity was observed but at an MIC value of 1.56 mg/mL for *Z. mucronata* across different boiling times [34]. The decreased activity observed among the decoctions in this study may be attributed to the prolonged boiling duration, which might have affected thermolabile phytoconstituents [35, 36]. Additionally, different geographical locations and harvesting seasons could also contribute to the observed discrepancy in the observed antigonococcal activity across the different plants tested [37, 38]. While the microdilution assay (incorporating INT as an indicator reagent) used to determine the MICs provides a reliable endpoint MIC determination, it does not account for the continuous quantitative data needed to generate dose‐response curves and IC_50_ computation. Future studies will incorporate microplate reader‐based assays for more detailed pharmacodynamic analysis.

There was no correlation observed between the percentage yield and observed antigonococcal activity. In some instances, high‐yield extracts like methanol and the decoctions (e.g., ZML5, 22.10%, MIC = 12.5 mg/mL) displayed poor antigonococcal activity, stressing the idea that yield doesn't directly translate to increased bioactivity [40]. Conversely, some of the extracts with low yield exhibited the highest antigonococcal activity, such as the dichloromethane and ethyl acetate extracts of *Z. rivularis* and *Z. zeyheriana* (e.g., ZRL2, 5.31%, MIC = 0.2 mg/mL; ZZR3, 0.43%, MIC = 0.2 mg/mL). These observations suggest that the observed antigonococcal activity is primarily driven by the presence of specific metabolites as compared to the overall extract yield obtained. Despite the promising antigonococcal activity observed and low in vitro cytotoxicity profiles across most extracts, the selectivity indices fall short of the desirable threshold (SI<10). The low SI observed suggests poor selectivity of the extracts to *N. gonorrhoeae*, suggesting potential cytotoxicity to mammalian cells [41]. An ideal drug candidate ought to demonstrate high selectivity (SI>10), indicating preferential toxicity to *N. gonorrhoeae* over the host cells. The low SI thus limits the therapeutic potential of these crude extracts. However, poor selectivity is common, especially at the crude extract level, and improvements are often observed with further bioassay‐guided fractionation and isolation of bioactive compounds [42]. Furthermore, the inactive extracts should not be interpreted as evidence of the absence of antigonococcal activity. Crude extracts are made up of complex metabolite mixtures in which synergistic and antagonistic interactions can mask or enhance the efficacy of the tested extracts [43]. As such, extracts that are inactive at the crude‐extract stage may later yield active fractions upon bioactivity‐guided fractionation and should not be prematurely excluded from further analysis. While the findings present significant antigonococcal bioactivity in vitro, further in vivo studies are needed to validate these under physiological conditions.

**FIGURE 1:**
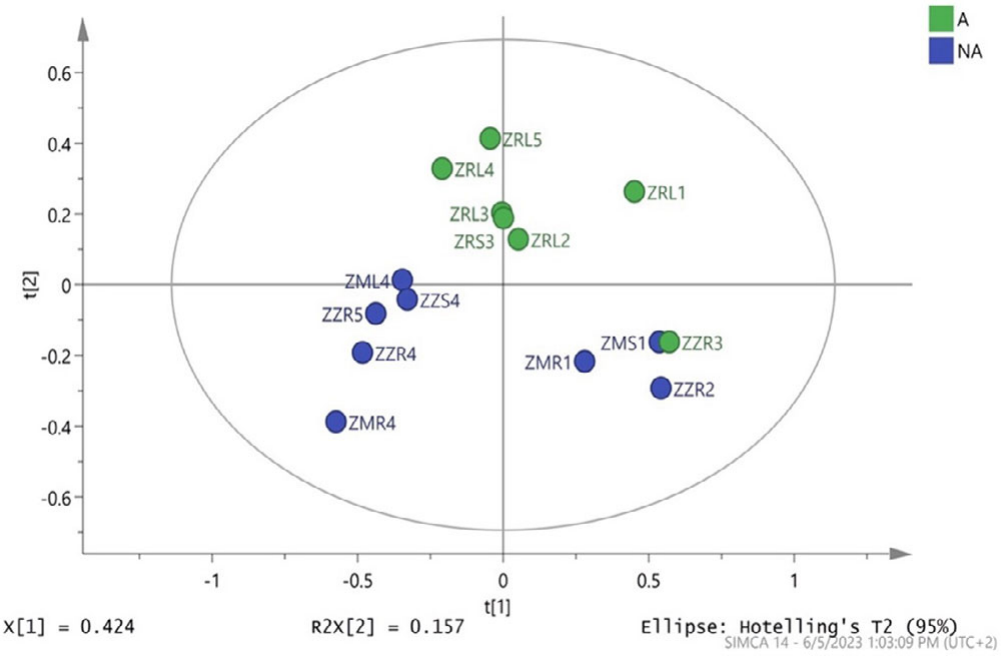
Principal component analysis (PCA) scores plot of 15 extracts from *Ziziphus* species indigenous to South Africa: (A)—active and (NA)—non‐active.

**FIGURE 2:**
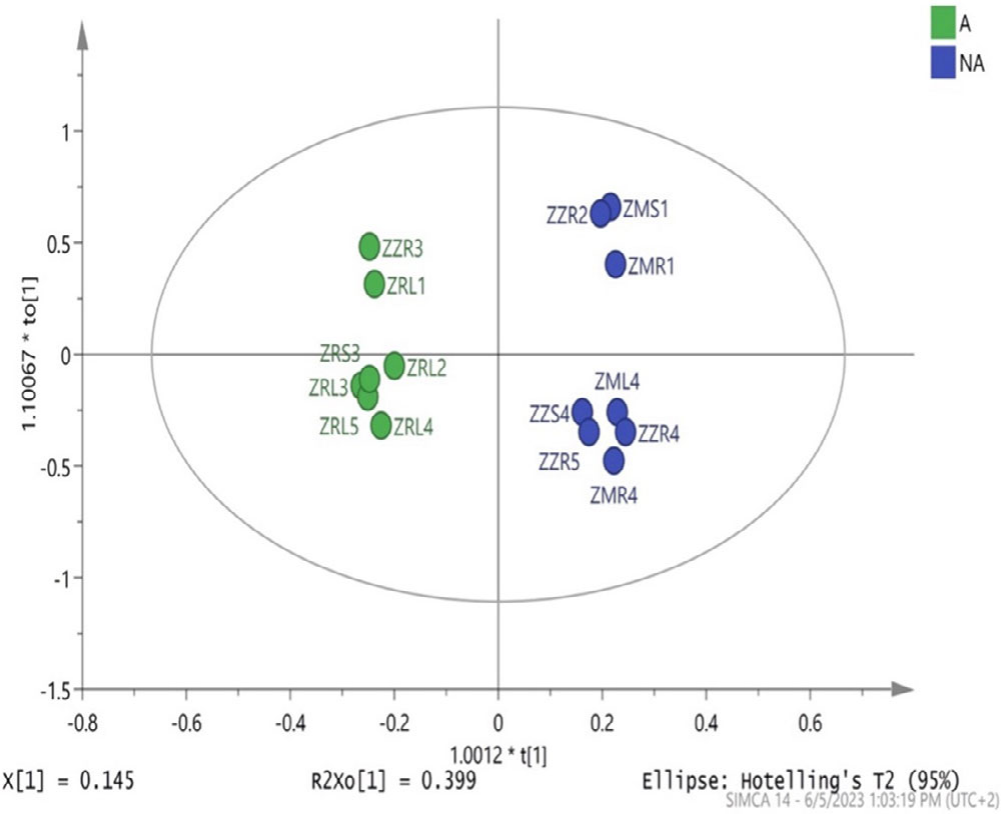
Orthogonal projections to latent structures‐discriminant analysis (OPLS‐DA) of 15 extracts from *Ziziphus* species indigenous to South Africa: (A)—active and (NA)—non‐active.

To further gain insight into the relationship between phytoconstituent composition and antigonococcal bioactivity, multivariate statistical analysis was conducted using principal component analysis (PCA). To minimize data skewing and improve the model's accuracy and robustness, extracts with MIC < 1 mg/mL were classified as active, while those with MIC values ranging from 1 to 3.13 mg/mL were deemed inactive. Extracts exhibiting an MIC > 3.13 mg/mL were excluded from analysis, resulting in a dataset of 15 samples that were subjected to ^1^H NMR‐based metabolomics profiling. The PCA scores plot displayed a general clustering pattern that distinguished between active and inactive samples of the training set (Figure 1). However, the ethyl acetate root extract from *Z. zeyheriana* (ZZR3; MIC = 0.2 mg/mL) clustered with the inactive group. The model produced *R* ^2^ and *Q* ^2^ values of 0.67 and 0.47, respectively. While the *R* ^2^ value indicates that the data fit perfectly in the PCA model, the moderate *Q* ^2^ value suggests limited capability for predicting antigonococcal bioactivity, especially when applied to unknown samples. Such limitations are suggestive of inherent variability within the metabolite profile and other limitations of an unsupervised PCA classification. To improve the discriminatory capabilities of the PCA model, the supervised orthogonal projections to latent structures‐discriminant analysis (OPLS‐DA) was applied. This model demonstrated a clear separation between active and inactive samples (Figure 2) and exhibited improved performance (*R* ^2^ = 0.69 and *Q* ^2^ = 0.77). The higher *Q* ^2^ value indicates improved prediction capabilities and model robustness, which was further validated by ANOVA cross‐validation (*p* = 0.05). The previously misclassified ZZR3 extract was correctly clustered with active samples, demonstrating the advantage of a supervised OPLS‐DA model in the resolution of sample overlapping in the PCA scores plot. Despite the improved classification observed in the OPLS‐DA model, there is some degree of in‐group variation observed, particularly among the inactive extracts. Such variability is attributed to the inherent chemical diversity within plant extracts, even in species of the same genus. When the ^1^H NMR spectra were processed, solvent peaks, water signals, and background noise were excluded for model clarity. Nevertheless, small phytochemical variations, especially among crude extracts, remain a significant challenge as related species often share major similarities in their metabolic profiles, further complicating discrimination solely based on spectral data [39].

**FIGURE 3:**
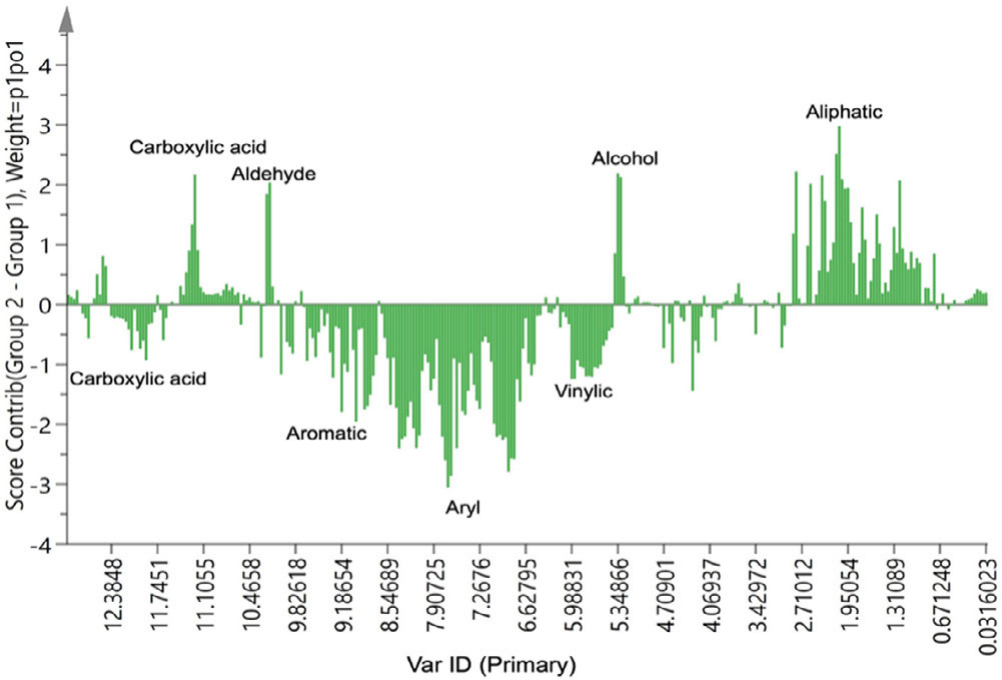
The contribution plot shows the major classes of compounds that can be attributed to the observed discrimination in the training set. (Active—bars projecting upward and non‐active—bars projecting downward).

**FIGURE 4:**
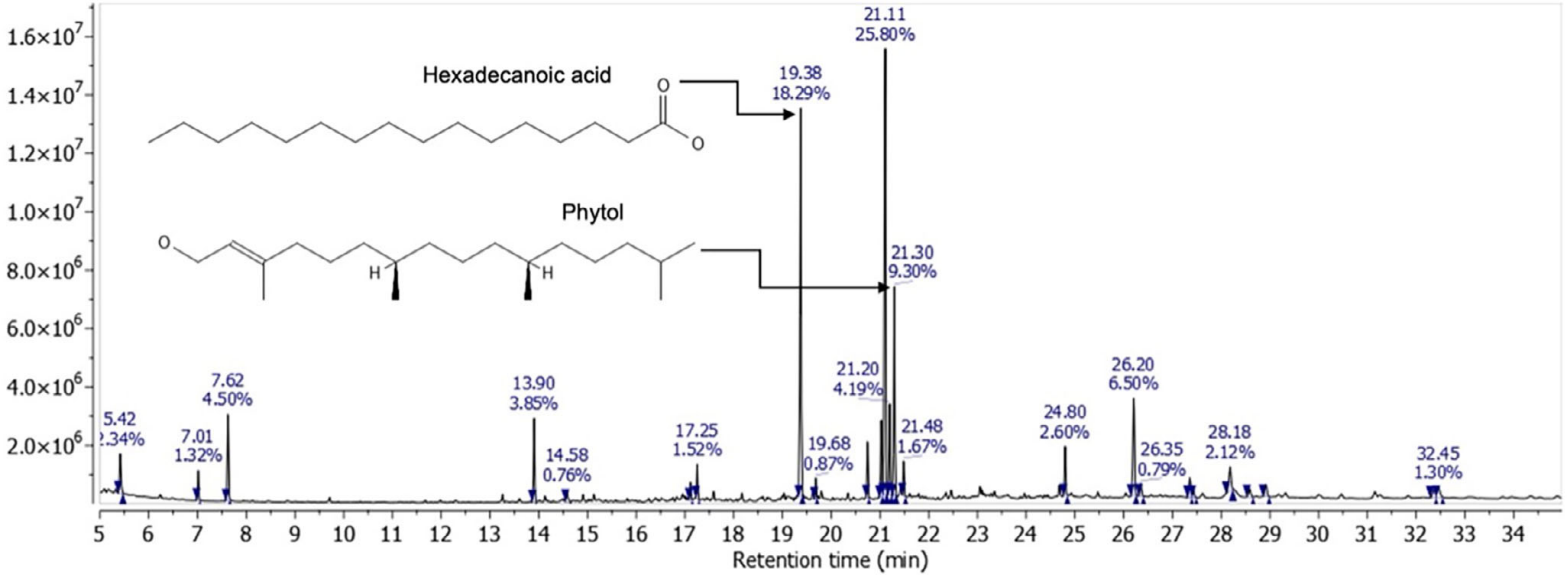
The total ion chromatograph (TIC) of the dichloromethane leaf extract from *Ziziphus rivularis* (ZRL2).

**FIGURE 5:**
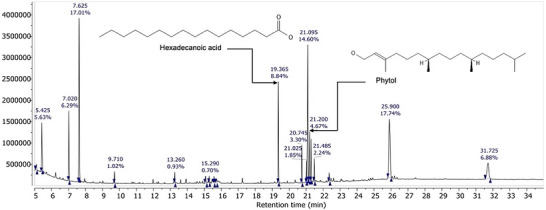
The total ion chromatograph (TIC) of the ethyl acetate:methanol (1:1) leaf extract of *Ziziphus rivularis* (ZRL4).

**FIGURE 6:**
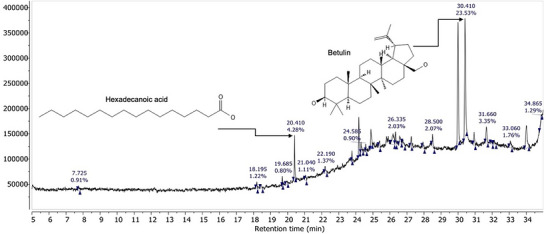
The total ion chromatograph (TIC) of the ethyl acetate root extract of *Ziziphus zeyheriana* (ZZR3).

Subsequent to the observed clear discrimination of the training set, a contribution plot was generated (Figure 3) to identify the specific chemical classes that may be associated with the observed antigonococcal activity. The classes tentatively linked to the observed antigonococcal activity include aliphatic compounds (δ 0–1.9 ppm), alcohols (δ 5.3–6.6 ppm), aldehydes (δ 9.8–10.4 ppm), and carboxylic acids (δ 11–12 ppm) (Figure 3). Conversely, certain chemical shifts in the carboxylic acid region suggested the presence of classes not contributing to the observed antigonococcal bioactivity. These results emphasize the complexity of plant metabolomes, particularly where specific morphological features within a chemical class may determine biological activity. To further understand the chemical basis of the observed antigonococcal bioactivity, selected extracts were subjected to GC–MS analysis. The dichloromethane leaf extract of *Z. rivularis* revealed the occurrence of hexadecanoic acid (Rt = 19.38 min) and phytol (Rt = 21.30 min) (Figure 4). Similarly, hexadecanoic acid (Rt = 19.37 min) and phytol (Rt = 21.20 min) were identified in the ethyl acetate:methanol (1:1; *v/v)* leaf extract of *Z. rivularis* (Figure 5), while hexadecanoic acid (Rt = 20.41 min) and betulin (Rt = 30.41 min) were tentatively identified in the ethyl acetate root extract (Figure 6). These peak sizes correlate to the relative extract concentration of 1 mg/mL, where larger peaks indicate a higher availability.

Although the identified phytoconstituents were not previously tested against *N. gonorrhoeae*, they have demonstrated antimicrobial biological activity against some clinical Gram‐negative bacteria. Hexadecanoic acid, for instance, displayed moderate activity at a maximum concentration of 50 mg/mL against *E. coli* and *Klebsiella pneumoniae*, exhibiting inhibition zones of 11.1 and 11.93 mm, respectively [44]. Phytol demonstrated significant antimicrobial activity against *E. coli*, *Salmonella*, and *H. pylori*, revealing inhibition zones of 10 – 20 mm. Further molecular docking of phytol on DNA gyrase revealed a good binding affinity, tentatively confirming its potential as an antigonococcal lead constituent [45]. Betulin, previously isolated from *Z. mucronata* root bark, displayed reduced antibacterial effects against *E. coli*; however, its derivatives exhibited increased activity from 24.0 to 0.015 mM [46]. The occurrence of these phytoconstituents in active extracts may suggest that they contribute either individually or synergistically to the observed antigonococcal activity. These phytoconstituents have demonstrated antimicrobial activity against Gram‐negative bacteria, similar to *N. gonorrhoeae*; nevertheless, additional testing, especially on *N. gonorrhoeae*, is necessary to fully assess their antigonococcal potential. Overall, these findings highlight that *Z. rivularis* and *Z. zeyheriana*, particularly the non‐polar and semi‐polar extracts, have significant antigonococcal potential. However, the low SI and the outstanding in vivo validation emphasize the need for further investigation, including bioassay‐guided fractionation, constituent isolation, and cytotoxicity evaluation. The integration of multivariate analysis and metabolomics provided valuable insights into the chemical basis of antigonococcal activity among *Ziziphus* species indigenous to South Africa. The application of a supervised OPLS‐DA was effective at discriminating between active and inactive samples, while the contribution plot and GC–MS analysis tentatively revealed key chemical classes as well as compounds behind the observed antigonococcal activity. Collectively, these findings offer a solid foundation for further bioassay‐guided isolation and subsequent inclusion into drug discovery initiatives.

## 3 Conclusions

This study has successfully validated the traditional medicinal use of *Z. mucronata*, *Z. rivularis*, and *Z. zeyheriana* in treating gonorrhea, with *Z. rivularis* and *Z. zeyheriana* showing antigonococcal activity for the first time. The low cytotoxicity observed in most of the extracts further reinforces the traditional applications of these plants in South African indigenous communities. It demonstrated the effectiveness of ^1^H NMR‐based metabolomics in accelerating the identification of potential bioactive chemical classes within *Ziziphus* species, using a robust OPLS‐DA model. The contribution plot generated from the OPLS‐DA model revealed chemical classes associated with the observed antigonococcal activity, setting the stage for future isolation and detailed phytoconstituent characterization. Notable compounds, including hexadecanoic acid, phytol, and betulin, were identified in *Z. rivularis* and *Z. zeyheriana* using GC–MS. Although previously reported to possess antimicrobial properties against some Gram‐negative bacteria, no studies have specifically tested their efficacy against *N. gonorrhoeae*, highlighting an area ripe for in‐depth research. Future research should focus on the bioassay‐guided isolation of active phytoconstituents to confirm their contribution to the observed antigonococcal activity in the crude extracts. Further structure elucidation and constituent quantification should be conducted to shed insights into the structure‐activity relationship. Given the limitations of in vitro assays, in vivo studies need to be conducted to establish the therapeutic efficacy of these isolated constituents in animal models.

## 4 Experimental Section

### 4.1 Plant Collection

*Ziziphus mucronata* plant samples were collected on January 5, 2022, from Limpopo Province in the Vhembe district Municipality (22°00'S; 29°12'E), while *Z. rivularis* and *Z. zeyheriana* samples were obtained on May 22, 2022 from the Manie Van Der Schijff Botanical Garden (25°45' S; 28°13' E) and the University of Pretoria Experimental Farm (25°00' S; 28°00' E), respectively. Ms Magda Nel performed all taxonomic identifications of the three *Ziziphus* plant species. Voucher specimens of the respective collected *Ziziphus* species were prepared and deposited in the HGWJ Schweickerdt Herbarium (PRU) under the following accession numbers: *Z. mucronata* (130883), *Z. rivularis* (130882), and *Z. zeyheriana* (0125140). The root, stem bark, and leaf materials were collected for *Z. mucronata* and *Z. rivularis*. For *Z. zeyheriana*, only the root and stem bark samples were obtained. Seasonal senescence prevented the collection of the leaves as they were not available. In total, eight plant samples were collected from the three species, air‐dried, and powdered using an Ultracfugal Mill (Retsch, Germany). The samples were then stored at room temperature (20°C–25°C) until further extraction experiments.

### 4.2 Plant Extraction

The dried and powdered plant material (40 g per sample) was extracted sequentially using a series of solvents with increasing polarity. The extraction solvents used were: (1) *n*‐hexane, (2) dichloromethane, (3) ethyl acetate, (4) ethyl acetate:methanol (1:1; *v/v*), and (5) methanol. For each extraction step, 400 mL of analytical‐grade solvent was added to the plant material, followed by homogenization in a blender (Philips, Pretoria) for 5–10 min. Subsequently, the texture was sonicated in an ultrasonic water bath (Labotec, Midrand) for 10 min and filtered through a Whatman No.1 filter paper (Merck, Germany). Each respective solvent extraction was performed in triplicate (three successive cycles) on the same plant material to ensure an exhaustive extraction before proceeding to the next solvent. The final combined filtrates for each respective solvent were concentrated under reduced pressure utilizing a rotary evaporator (Buchi, R‐200, Switzerland) and then transferred into pre‐weighed glass vials. In addition to the organic solvent extractions, aqueous decoctions were also prepared to mimic traditional preparation methods [47]. For this, 40 g of each plant sample was boiled in 500 mL of tap water at 100°C for 45 min [48]. The resulting mixture was cooled in an ice‐water bath, frozen at −80°C overnight, and subsequently freeze‐dried using a manifold freeze dryer (Virtis, New Zealand) to obtain dried extracts. In total, 48 successive extracts were obtained from the combined sequential extraction and decoction procedures.

### 4.3 Bacterial Culturing

*N. gonorrhoeae* (ATCC 49226) was maintained on chocolate agar (Sigma‐Aldrich, Missouri, USA) and cultured in Mueller‐Hinton (MH) broth (Merck, New Jersey, USA) for a 24‐h incubation period following the procedure described by Wiegand et al. and Klančnik et al. [49, 50]. For inoculum preparation, a single colony was transferred into 50 mL MH broth and further incubated for 20 h at 37°C in a shaking incubator (Labotec, South Africa). To provide a CO_2_‐enriched atmosphere required by the *N. gonorrhoeae* to grow optimally, the bacterial cultures were incubated in an airtight container containing CO_2_‐generating sachets to create an anaerobic environment (Thermo Scientific, South Africa). After the incubation, the bacterial suspension was standardized to an optical density (OD_600_) of 0.2 using sterile MH broth and a spectrophotometer (Labotec, South Africa). To avoid contamination and maintain optimal sterility, all procedures were conducted in a sterile biosafety cabinet (ESCO, Singapore). All experiments were conducted as two independent assays, with each assay conducted in triplicate (n = 3). The results are expressed as the mean of the combined replicates.

### 4.4 Minimum Inhibitory Concentration (MIC) Assay

The minimum inhibitory concentration (MIC) of all 48 successive extracts was determined using the broth microdilution assay, a cost‐effective, reliable, and reproducible method. This approach is best suited, especially when assessing the microbial effects of plant extracts [49, 50]. Briefly, 50 mg of each extract was dissolved in 1 mL of 10% dimethyl sulfoxide (DMSO) to obtain a 50 mg/mL stock solution. Ciprofloxacin (Merck, Modderfontein) served as the positive control, while the negative control consisted of 10% DMSO and broth (1:1, *v/v*). Twofold serial dilutions of each extract were prepared to yield final concentrations ranging from 12.5 to 0.01 mg/mL. Two independent assays were conducted, with each extract tested in triplicate (n = 3) in 96‐well ELISA microtiter plates (Sigma‐Aldrich, Missouri, USA). After incubation, bacterial proliferation was assessed by adding 40 µL of *p*‐iodonitrotetrazolium (INT) solution (Sigma, Missouri, USA) followed by a further 2‐h incubation. Development of a pink color indicated bacterial proliferation, whereas the absence of color change indicated inhibition of bacterial growth. The MIC was defined as the lowest concentration at which no visible color change was observed in the microtiter plates. The antigonococcal activity was classified according to MIC values, with extracts exhibiting MIC < 1 mg/mL considered highly active, values between 1 and 6.25 mg/mL were moderately active, and values above 6.25 were considered inactive [51].

### 4.5 Cytotoxicity Assay

The cytotoxicity of the 48 successive extracts was tested for cytotoxicity in vitro using rat skeletal myoblast (L6) cells (CVCL_0385), procured from Banco de Células do Rio de Janeiro (BCRJ, Brazil; Cat. no. 0141). Podophyllotoxin (Sigma‐Aldrich, P440) was used as the positive control. The cells were seeded in 96‐well ELISA microtiter plates at a density of 4 × 10^3^ cells per well in 100 µL of RPMI 1640 medium supplemented with 10% fetal bovine serum (FBS) and 1% L‐glutamine and further incubated under standard cell culture conditions. Eleven threefold serial dilutions of the respective extracts were prepared to yield final concentrations ranging from 100 to 0.002 µg/mL. This was followed by a 70‐h incubation period, cell morphology, and confluence examination using an inverted microscope. Cell viability was then assessed by the addition of resazurin solution to a final concentration of 10 µg/mL, followed by another 2‐h incubation. Fluorescence was measured at excitation and emission wavelengths of 536 nm and 589 nm, respectively, using a SpectraMax Gemini XS microplate fluorometer (Molecular Devices, California, USA). The required concentration to inhibit 50% of viability (CC_50_) was determined by non‐linear regression of sigmoidal curves. Cytotoxicity was classified as cytotoxic (CC_50_< 0,05 mg/mL), moderately cytotoxic (CC_50_ between 0.05 and 0.1 mg/mL), and non‐cytotoxic (CC_50_> 0.1 mg/mL) [52].

### 4.6 Nuclear Magnetic Resonance and Multivariate Data Analysis

All 48 extracts were subjected to ^1^H NMR analysis to compare the chemical profiles of the extracts based on their chemical profiles. Each extract was dissolved in 700 mL of deuterated dimethyl sulfoxide (DMSO‐d_6_ to a final concentration of 15 mg/mL. The solutions were then vortexed to dissolve all particulates and then transferred into 5 mm NMR tubes (Sigma‐Aldrich, Missouri, USA). ^1^H NMR spectra were recorded at 25°C on a 400 MHz Varian NMR spectrometer. For each sample, spectra were acquired over a spectral width of 0–14 ppm using 64 scans with standard acquisition parameters. Before the data acquisition, the magnetic field homogeneity was optimized by manual shimming to ensure consistent spectral resolution across all samples. The spectra were processed using MestReNova software (Version 14.20, Mestrelab). Free induction decays (FIDs) were Fourier transformed, and the resulting spectra were then manually phase‐corrected and automatically baseline corrected using the Whittaker smoothing algorithm [53, 54]. Chemical shifts were referenced to the residual solvent signal of DMSO‐d_6_ (δ 2.50 ppm). For multivariate data analysis, all processed spectra were uniformly binned into regions of 0.04 ppm over the δ 0.00–14.00 ppm range. Regions on the spectra that correspond to the residual solvent signal (δ 2.40–2.60 ppm) and residual water signal (δ 3.20–3.40 ppm) were excluded before further analysis. The binned data were subsequently exported as ASCII files and then imported into SIMCA‐P software (version 14.1, Umetrics, Umeå, Sweden) for multivariate statistical analysis. ^1^H NMR data acquisition, processing, and multivariate analysis were performed according to established NMR‐based metabolomics protocols for plant extracts, including spectral preprocessing (phase and baseline correction), binning, and statistical analysis using SIMCA‐P [54].

### 4.7 Gas Chromatography–Mass Spectrometry (GC–MS) Analysis

Three extracts exhibiting the best antigonococcal activity (MIC < 1 mg/mL) were selected for GC–MS analysis to tentatively identify compounds attributed to the observed bioactivity in *Z. rivularis* and *Z. zeyheriana*. The successive extracts analyzed included the dichloromethane leaf extract of *Z. rivularis* (ZRL2), the ethyl acetate:methanol (1:1) leaf extract of *Z. rivularis* (ZRL4), as well as the ethyl acetate root extract of *Z. zeyheriana* (ZZR3). Each extract was resuspended in the respective extraction solvent before the analysis. Derivatization was performed using *N,O*‐bis(trimethylsilyl)trifluoroacetamide (BSTFA) at room temperature for 5 h to produce trimethylsilyl (TMS) derivatives, thus enhancing the volatility and thermal stability of the polar metabolites. The GC–MS analysis was conducted as previously described by Mabuza et al. with minor modifications [27]. Briefly, 1 µL of each derivatized extract was injected in a split mode at an injector temperature of 250°C into a Shimadzu GC–MS (QP2010 SE, Shimadzu, Kyoto, Japan) equipped with an Rxi‐5MS capillary column (30 m × 0.25 mm, 0.25 µm film thickness; Restek, Bellefonte, PA, USA) composed of 5% diphenyl/95% dimethylpolysiloxane stationary phase. The oven temperature was set to an initial temperature of 60°C (held for 3 min), followed by an increase to 250°C. The oven was programmed to 60°C, held for 3 min, and then increased to a final temperature of 280°C at a rate of 15°C per minute. While the ion source temperature was set at 250°C, the interface temperature was also set at 250°C, at 15°C/min. The ion source and interface temperatures were maintained at 250°C. Mass spectra were acquired in electron ionization (EI) mode at 70 eV over a mass range of m/z 30–550 with a 2000 scan speed. The tentative identifications of the phytoconstituents were conducted by comparing the obtained mass spectral fragmentation patterns with those in the NIST 11 and Wiley 09 mass spectral libraries.

## Author Contributions

**Mcebisi Junior Mabuza** conceptualized the study, conducted formal analysis, methodology, and visualization, and wrote the original draft. **Marcel Kaiser** assisted with the biological activity screening and cytotoxicity. **Mahwahwatse Johanna Bapela** provided supervision, resources, and proofreading of the manuscript. **Thilivhali Emanuel Tshikalange** provided resources and supervision and assisted with reading the original draft manuscript. **Abdullahi Ahmed Yusuf** assisted with GC–MS analysis and proofreading the manuscript.

## Conflicts of Interest

The authors declare no conflicts of interest.

## Data Availability

The data that support the findings of this study are available from the corresponding author upon reasonable request.
